# First Examination of Mrs. Cumming by the Commissioner. January 13th, 1852

**Published:** 1852-04-01

**Authors:** 


### FIRST EXAMINATION OF Mrs. CUMMING BY THE COMMISSIONER.

January 13th, 1852.

Q. You have not seen so many people for a long time? A. No. — Q. Do you remember seeing me in the year 1846—it is some time ago? A. I have not forgotten you. — Q. This is a better house than you had at Belgrave-terrace, or street? A. Yes, it is. — Q. We went and saw that? A. I recollect your going to see it. I requested they would let you see it. — Q. You had some cats there. I do not remember how many? A. Yes, I had cats. — Q. I think you had pigeons there? A. Yes, I had: if it was any mark of insanity to keep pigeons, there are a good many people who would be taken to the madhouse. — Q. This is a much more comfortable place than that was, as far as I can judge? A. Yes. — Q. This is your own house, I think? A. This is my own house. — Q. You bought it? A. Yes, I bought it. — Q. And the adjoining house? A. You are right. — Q. Do you know what you gave for it? A. Yes, I do. — Q. I am afraid I must ask you what may appear to be an impertinent question to a lady, but I hope you will not be offended? A. If you do not ask me too many. — Q. What did you give for it — do you remember? A. Do you mean for the two? — Q. Yes, for the two. A. Why, I gave about 500*l.* — Q. Do you know what the ground-rent is upon either of them: you know that to the person who owns the soil you pay a ground-rent—do you know what it is? A. I could not pay all the money that was required for it, because there was a mortgage of it. — Q. Were you to pay 500*l.* besides the sum that was mortgaged. I will try and explain to you, in order that you may not misunderstand me—you think you paid 500*l.* for it, but there is a mortgage? A. Yes, there is a mortgage. — Q. Do you know the amount? A. Yes, I do. — Q. How much? A. I do not know that I am authorised exactly to expose my private affairs in that way: do not deem me impertinent to you, Mr. Barlow. — Q. No, I will not; that is why I apologised for asking you the question: these gentlemen are on their oaths, and therefore I am bound to ask you the questions which are suggested by them. You would rather not say how much? A. I had rather not. — Q. We have been told the value of the houses with the mortgage—how much do you think it worth with the mortgage. You were to give 500*l.* for it? A. Yes; there are two houses, you know; I could not give the whole amount—not the whole value of the houses. — Q. But there is a mortgage? A. There is a mortgage. — Q. You would rather not? A. Divulge it. — Q. But I suppose if the interest were not paid on the mortgage you would be troubled by the persons to whom the money is owing? A. But the interest *I* paid. — Q. By whom? A. By my solicitor—by my desire. — Q. May I ask the amount of money—the interest that is paid? A. Why, I consider, Mr. Barlow—I consider it is a private affair, and as I am persecuted so much about my property, I think it right to keep those affairs in my own breast—do not deem me impertinent in giving that answer. — Q. I understand you to say that you have been persecuted; what makes you think you have been persecuted—who do you think have persecuted you about your property? A. My two daughters. — Q. Anybody else? A. No, not now. — Q. You mean Mrs. Ince and Mrs. Hooper? A. And Mrs. Hooper: they have most grossly persecuted me; wherever I go to, I am persecuted by them the moment they find me out. — Q. In what way do they persecute you? A. By coming to my house and annoying me, and putting me in the position I am placed in at present when they get at me. — Q. This house is a very comfortable one, apparently? A. Yes, but I am not speaking of the house, but I am speaking of the inquiry that is taking place with regard to my sanity. — Q. You mean they took out a commission in 1846? A. Yes, they did. — Q. Had not Mr. Cumming something to do with it before? A. Ah, he had—it was so nominally—they said he did it. — Q. What kind of person was Mr. Cumming—did you live quietly and happily together? A. We did. — Q. I ask you the question at the suggestion of your own friends, and others—was he a person quiet, and of good temper—I am told you treated him with kindness sometimes, and sometimes not? A. He is dead, and let all faults be buried with him. — Q. I believe you told me that before? A. I did. — Q. Was he a free living gentleman with ladies; do you remember, because there is some allegation about his nurses? A. He had some very bad nurses. — Q. Are you quite sure of that from your own personal observation? A. Yes. — Q. Did you ever see it yourself, personally? A. I did. — Q. You had no doubt about it in your own mind? A. No, because I had ocular demonstration of it. — Q. I do not like to ask you about that more minutely? A. I could not enter into it for decency's sake—decency would not allow me. — Q. But it was of that kind that you refer—it was of an indecent character? A. Yes; decency forbids me to mention it. — Q. Was that shortly before he died that you refer to? A. Yes. — Q. During his last illness? A. I was not with him. — Q. Was it shortly before you went away? You were taken away some little time before he died? A. Yes; it was shortly before he died. — Q. Now, this mortgage about these two houses—you do not know the precise amount—you do not wish to tell us the precise amount? A. I do know it certainly, but it is an affair I should not like to enter into. — Q. The next house you let to somebody? A. Yes, it is let. — Q. Do you know what rent is paid for it? A. Yes. — Q. Have you received it yourself? A. I have seen the house, but have never been all over it. — Q. Who receives the rent for you? A. Mr. Robert Haynes. — Q. How long ago is it since you bought these houses? A. I do not know. — Q. I do not think we have been told that? A. It is now seven years ago, I think. — Q. Since I had the pleasure of seeing you at the Horns? A. Oh, yes; what is that. — Q. Do you remember the year. I saw you on the other side of the water—that was in 1846? A. Yes, in 1846. — Q. How long was it after that that you bought it? A. A good bit. — Q. Has that adjoining house been let ever since? A. Yes, to the same ladies who took it. — Q. Have they ever paid you the rent yourself? A. No; I never demanded it of them. — Q. Who is it paid to? A. Mr. Robert Haynes. — Q. And he can tell us all about it? A. He can tell you all about it. — Q. Has he ever sent you an account of his receipts for it? A. Yes; he has given me an account of it. — Q. From time to time? A. Yes, from time to time. — Q. Do you know what the rent is? A. Yes. — Q. What is the amount of it? A. Yes; the amount of the rent yearly. — Q. What is it: it is a smaller house than this, judging of it from the outside? A. It is much the same, but not so well finished a house as this. — Q. What is the rent of it—what do you get for it, I mean—do you remember what it is a year? A. I do; but my daughters make all inquiries to know how much this estate is, and how much the other, and that makes me more reserved now. — Q. But I do not come here by order of your daughters? A. No; I know you do not. — Q. You do not wish to tell us? A. No; most certainly I do not. — Q. But Mr. Haynes has received it always for you? A. Yes. — Q. What other property have you got? A. Do you mean in London or Wales? — Q. Have you any in London besides those two houses? A. No, not besides. — Q. But you have some near Newport? A. Yes, in the neighbourhood of Newport. — Q. Do you know what it produces you a year? A. Yes. — Q. How much? A. Why, I have sold some of it, you know. — Q. We are told you have sold it? A. I sold it, and sold it very advantageously; and the other I was obliged to sell to the Railway Company—of course, it was a matter of obligation—it was not a matter of choice, that was not. — Q. Do you know what you got for the bit you sold to the Railway Company? A. Yes. — Q. How much? A. I have only got this [referring to a paper] to tell you, in case you do not know. Do you know what you were to have for that from the Railway? A. Which of the houses? — Q. That I cannot tell you; you sold one property to the Railway Company, and another to the Waterworks Company? A. You are right. — Q. Do you remember what you were to get for that which you sold to the Railway Company? A. There were two properties sold to the Railway Company. — Q. And one to the Waterworks Company as well? A. Yes. — Q. What did you get for each of those which you sold to the Railway Company—do you know?

K

A. Which do you allude to? There is one in Newport and one in Bassaleg. — Q. What were you to have for the one at Newport? A. I would not let the house without the grounds, because it was built for a gentleman's house, and of course no gentleman would have taken the house, the railway passing by it. — Q. What were the railway to give you for the house and for the grounds? A. They were to give me for the grounds and for the house 2000*l.* — Q. 2000*l.* value? A. Yes. — Q. For the house and grounds, you mean, at Newport? A. Yes. — Q. What were they to give you for the other? A. Bassaleg? Q. Yes? A. They were to give me 2000*l.* for it: that is, 4000*l.* — Q. Then for the Waterworks, they took something, did they not? A. It is made the Waterworks now. — Q. The Newport Waterworks Company? A. They bought it, you know. — Q. That is the third sale? A. That is the third sale. — Q. What were they to give you for it? do you recollect? I am afraid I cannot assist you in it. Do you remember what they were to give you for it? A. Yes, they were to give me 3000*l.*, that is, 9000*l.* altogether. — Q. No, no? A. No, very near it. Is it not very near it? — Q. The first 2000*l.*, do you know what has become of it; have the railway paid it to you? A. Yes. — Q. Did they send you the money—did they send it to your bankers? A. Yes, they did; at least it came to London. — Q. They paid it into court, I suppose? A. They did not pay it into court; they paid it here, at the attorney's office. — Q. Is that Mr. Haynes? A. Yes. — Q. He received it as your solicitor? A. I was present when he received it, and he handed it over to me. — Q. Did he hand it over to you in the shape of money? A. Yes, he did, and then, you know, I had a great number of debts to pay, and amongst them my law expenses. — Q. That was money they handed over to you. They came up to London and paid you that? A. Yes. — Q. Now, the other 2000*l.*, which I suppose was in some way or other in the Court of Chancery—I do not know? A. No. — Q. Do you know what became of the other 2000*l.*? A. Yes, most of it, indeed all of it, was paid in law expenses, the 3000*l.*; it cost me that or thereabouts, you know; it is many years ago now. — Q. In 1846? A. In 1846. — Q. Did they send you an account of those law expenses? A. They showed me an account. — Q. They showed me a paper the other day—I do not know whether that is the one they showed you—in which the sum was, I think it was between 600*l.* and 700*l.* only. It was an account that was sent to you; there was a list of witnesses at the side, and an abstract of the payments at the end. Do you remember that being shown to you ever? A. Yes, he always brings every account of any monies he has had. — Q. Who always does? A. Mr. Haynes. — Q. Have you confidence in him? A. The most implicit confidence. — Q. Do you remember when you first saw him? A. Remember when I first saw him—what? — Q. How came you to be acquainted with him? A. In the regular routine of things. I met him. I was introduced to him by some friends that I had. — Q. Do you know who they were—I am talking of Mr. Haynes, you know? A. I know. — Q. It is some years ago? A. It is a good many years ago? — Q. Before 1846? A. When I got acquainted with him? You know I visited him. I was introduced, regularly introduced. — Q. To Mr. Haynes? A. To Mr. Haynes. — Q. Where did he live then? A. He lived where we are now. — Q. In this house? A. In this house. — Q. Did he sell you this house? did you buy it of him? A. I bought it of him. — Q. He can tell us, though you cannot, what is the mortgage upon it? A. He can tell you. — Q. But you cannot recollect when you first saw him, or how you became acquainted with him? A. I was brought up to his house by some friends, I think, and I spent the evening here. — Q. Do you remember who it was who brought you here? A. Yes, I do remember. — Q. Who were they? A. I did not know that I was obliged to — Q. I will not oblige you to say anything that you do not like; but still, these gentlemen suggest that I should put questions to you, and they will draw their own inference if you do not answer them; but, at the same time, I am bound to inform you that you are not compelled to answer any question which is disagreeable to you. Do you think you knew him before or after 1846? A. It was after my husband died that I met him. — Q. You did not know him till after Mr. Cumming died? A. No. — Q. Who was your solicitor in Mr. Cumming's lifetime, do you remember? A. Why, Mr. Stone was my solicitor. He is dead now. — Q. Was not Mr. Dangerfield? A. Never. — Q. Was he not concerned for you? A. He never was; never as my solicitor, but he has done business for me as solicitor, because Mr. Stone unfortunately died, and my silver plate, and my deeds, and all my papers were transferred over to Mr. Dangerfield. — Q. Do you remember when it was that Mr. Stone died? A. Some years ago, now. — Q. And you think it was handed over from Mr. Stone to Mr. Dangerfield? A. I do not *think* it, I speak positively of it. — Q In consequence of Mr. Stone's death? A. In consequence of Mr. Stone's death. — Q. Mr. Cumming was alive then? A. Yes, he must have been. — Q. Did not Mr. Dangerfield look after some property for you at Newport, and appoint an agent for you down there? A. No, he did not. — Q. Was not Mr. Hawkins once an agent? A. Yes, he was. — Q. You dismissed him? A. I had just reason to do so. — Q. Then Mr. Dangerfield? A. Was acting, you know, for Mr. Stone; at least he could not act for him, because Mr. Stone was dead. — Q. But you saw him and let him act for you? A. Yes. — Q. Do you remember a report he made that was sent to you of the property in the neighbourhood of Newport? A. I have not read it. — Q. Do you remember seeing a report of that kind made by his brother? A. It might be by his brother; he never was my solicitor originally.

A Juryman.—Would you allow the ladies to retire, and let Mrs. Cumming be alone in the room with us?

The Commissioner (addressing Mrs. Cumming).—You do not mind their going away? I will take care of you; if you want anything let me know—Dr. Caldwell is here. [Two females who had been present up to this time retired.]

A Juryman.—I would rather the ladies left the room, because I saw one of the ladies looking at her.

Mrs. Cumming.—They were not making any motions to me to teach me what to say; they are in a rank in society above that.

The Commissioner.—No; only if they are out of the way they cannot be accused of it, you know. I always like to prevent the possibility of there being any mistake. — Q. You cannot tell how Mr. Haynes has applied these sums of 2000*l*., 2000*l*., and 3000*l*., can you? A. Yes. — Q. Has he bought any other property with it? A. He has paid a great many law expenses for me, and I have had something to live on, for I have nothing but my income. I am in no way of business at all. — Q. What is the income of this property that you have in Wales? A. You will excuse me for not answering that question—it is not out of any disrespect to you, Mr. Commissioner Barlow. — Q. You get money from time to time. How have you, for the last two or three years, been receiving your rents? A. In the regular way that any other person would—through agents. — Q. Who has been your agent—Mr. Haynes? A. Sometimes he has, and sometimes he has not. — Q. Was Mr. Thorne ever employed? A. No, never; for I would not trust Mr. Thorne to cross that table. — Q. You knew nothing about him? A. No; but I know too much about him. — Q. What has he done—in what way has he ever offended you? Did he not attend to your business? A. I was going to put my business into his hands, but I thought, after the little I saw of him, the wisest way was not to trust him any more. — Q. In what way did he give you personal offence? A. Why, sir, I asked him to call upon Mr. Haynes and ask him for some money, not being aware at that time that Mr. Robert Haynes had not received the money, which would have accounted for it, and I could get no satisfactory answer, and then I asked Mrs. Hutchinson's husband to be kind enough to call upon him and see him personally. — Q. To see Mr. Haynes? A. To see Mr. Haynes personally, which he did; and I have an answer—a satisfactory one, and he showed the accounts between us, you know. — Q. Mr. Haynes did? A. Mr. Haynes did to Mr. Hutchinson. — Q. But did he show them to Mr. Thorne, do you think? A. I do not think he did; I cannot tell. — Q. You applied to Mr. Haynes for his accounts, and he sent you a copy of them? A. He kept a copy. — Q. Did you send that to Mr. Thorne? A. No. Mr. Thorne took the papers himself, with a will. — Q. I will speak to you about that presently. Did you not send Mr. Haynes' account on to Mr. Thorne? A. No. — Q. Mr. Haynes told you he had sent you an account of his receipts to some particular year (I may be wrong); and did you not send that on to Mr. Thorne? A. The receipts to Mr. Thorne? — Q. No, the accounts to Mr. Thorne? A. No. — Q. You did not send it on, you think, to Mr. Thorne? A. No. — Q. Because Mr. Thorne produced this, and said he received it from you? A. I dare say he said so. — Q. With a letter from you to him (Thorne); you do not remember anything about it? A. I remember sending him a letter telling him I declined any more of his interference. — Q. He never sent you any bill of costs? A. I do not know what it could be for. — Q. This (referring to a paper) is an account which it seems you forwarded to Mr. Thorne, and I was going to ask you about one or two figures in it. Do you remember sending it on to Mr. Thorne? A. I do not remember anything of the kind; he has had it, perhaps, from Mr. Robert Haynes. — Q. No, he says he had it from you—you do not recollect it? A. No, I do not recollect it. — Q. Do you remember writing to Mr. Thorne? A. I remember writing to him—I declined his services. — Q. Do not you recollect any other letter? A. He took the will and several other papers away with him. — Q. When you wrote letters to Mr. Thorne did you write them yourself, or did anybody write them for you? A. I wrote them myself. — Q. Are you in the habit of writing letters yourself, or do you get your friends to write them for you? A. No; I do not get my friends to write them for me. — Q. Do you not get people to write letters for you and then sign them yourself? A. I may have done so sometimes. — Q. I see there is a sum here of 79*l.*, which I understand you say you never received; do you remember anything about that? A. Seventy-nine pounds which I never received from Mr. Robert Haynes? — Q. Yes. — A. He sent it over to me. — Q. Did you get it? A. I got it. — Q. Was it not sent by the coachman? A. It was brought by the coachman. — Q. Do you remember in what shape it was—was it a cheque, or bank note, or gold, or silver?

A Juryman (addressing Mrs. Cumming).—May I poke the fire—I am afraid it will go out?

Mrs. Cumming.—If you please.

The Commissioner.—He has not known you for seven years, but still I suppose he may do it?

Mrs. Cumming.—If he will take the trouble.

The Commissioner.—You had that 79*l.*? A. Yes. — Q. There seems to be some doubt whether you had it yourself—whether it got safe to you? A. Yes; I received it from the coachman. — Q. Do you know whether it was in notes, or what? A. In notes, I think. — Q. Did you not tell Mr. Thorne that you never had received it? A. I had not received it then. — Q. Not when you saw Mr. Thorne? A. No. — Q. Can you tell us when you did receive it? A. Oh, after that. — Q. It is a large sum to have received through a coachman. I should not like to trust my coachman with 79*l.*; but you are more liberal with your people—you think you had not received it at this time? A. I did receive it. — Q. Can you tell us when? A. No; I really do not know. — Q. You cannot recollect about when it was you received it? A. I suppose it was about nearly the time I received some of my rents; I should think so, but not being aware that I was to be called to account about my own property, I certainly have not a memorandum of it. — Q. I will tell you why I ask. We have been told that you have seen this account, and that you told Mr. Thorne you never received it; but now you seem to intimate—I do not know whether rightly or not—that you received it afterwards. A. Afterwards. — Q. After when—after this account was delivered? A. Did Thorne tell you that I gave him this? — Q. Yes, he did. A. Then he told a falsity. — Q. I will tell you what Mr. Thorne said—that he received a letter from you —signed by you; I do not remember the date, enclosing this, and that he then went to Mr. Haynes, and asked for an explanation as to the 79*l.* A. Yes. — Q. When you sent that to Thorne had you received that 79*l.*? A. If I had received it I should not have named it. I need not have applied to Mr. Thorne for money if I had received that sum, because it would have lasted me some little time if I had received a sum as large as that, you know. — Q. What kind of sums has Mr. Haynes paid you from time to time—does he pay them to you in large sums or small? A. Sometimes large and sometimes small. — Q. What is the general amount of the sums he pays you—does he pay the rents over to you or to your banker, on your account? A. He gives me money. — Q. You have a banker's account, have you? A. Yes, I have. — Q. Does he never make any transfer into your banker's account, because that is a more convenient way? A. He did down in Wales, but that man has broke. — Q. Who has broken? A Williams, the banker. — Q. Broke lately. A. Yes, not long ago.

A Juryman (to Mrs. Cumming).—There are no coals; may I ring for some?

Mrs. Cumming.—Yes, if you will take the trouble to ring.

A Juryman.—This bell rings?

Mrs. Cumming.—Yes, both of them.

(A bell is rung and a man servant enters).

Mrs. Cumming.—Some coals, George.

(The man puts some coals on the fire.)

Mrs. Cumming.—Have you got a wheel there? The fire has got so very low, that I wanted him to bring a wheel.

The Commissioner.—You do not generally sit in this room, do you—you usually sit upstairs? A. No, but I have a fire in it. — Q. You generally sit in the room upstairs? A. When I am not able to come down. — Q. Are your cats there now? A. No. — Q. Are they in the kitchen? A. I suppose they are; I very seldom go into the kitchen. — Q. When did they cease to live upstairs? A. Sir, they never lived upstairs—never; and whoever told you that, told you a gross falsity. — Q. Did they not live in your bedroom very much? A. They came up and down to have their meals, and then they were sent down again. — Q. Did they only come up for their meals? A. Yes, exactly so. — Q. Do you not remember, when the inventory was being taken of this house, some moisture having come through the ceiling? A. Not from the cats? — Q. From something else. A. The servants can best account for that. — Q. Was Mary Anne Hickey here, the little girl, at that time? A. She never lived with me as a servant; she only came with her mother. — Q. Did she not come here to reside? A. Yes. — Q. You paid her no wages, did you? A. Yes, I paid her wages. — Q. What did she come here for? A. To help occasionally the servant. — Q. Did she read the newspaper to you? A. Yes. — Q. And looked after these cats, for which you seemed to have some affection? A. No, she never looked after the cats, for I would not have trusted her. — Q. Do you remember that evening when something came through the ceiling, and you sent her up stairs to look at it. (No answer.) — Q. You went away from here some time ago, and since that you have been at Brighton. Before you quitted this house had you no cats in your bedroom? A. Never; not always. — Q. Were they there at night? A. No, only now and then coming backwards and forwards when the servant left the door open; the cats would naturally come up. — Q. But you did not wish them to go up? A. No. — Q. They did not dwell there night and day? A. No. — Q. You don't remember about this 79*l.*—you think you had it? A. I know I had it. — Q. Can you tell me when? A. I certainly did not make a memorandum of it, because I was not aware that I should have been called to account by any one as to what I had done with my own money. — Q. But you had it through the coachman? A. Yes, I had it through the coachman. — Q. This (referring to a paper) is an order in Chancery in a suit in 1848 about some money being paid to Mrs. Hooper, and in another part being paid Mr. and Mrs. Ince, and I see, at the end of it, there was an order for a sum of 2000*l.* You may read it if you like. I do not know that you ever saw that paper before? A. No. — Q. The 2000*l.*, by an order on the petition of the railway company, was to be paid to the credit of the cause—should be paid to the defendant, Catherine Cumming, and that the purchase money payable by the Newport Waterworks Company should also be paid to her. That is why I asked you about the railway company.—Do you think you sold property to the amount of as much as 2000*l.*? A. There was one for 3000*l.* and another for 3000*l.*, that is 6000*l.* — Q. And what was the other? A. And there were two small farms that were sold. — Q. That is a different thing. I am going to take the liberty of asking you about that presently. There was some sold to the Waterworks Company. A. I sold that for them to do as they liked with. — Q. They have the right to take it from you, under the act of parliament —you could not help yourself? — A. Not that property, but what was taken for the railway was under the act of parliament. — Q. What did you get for that which you sold to the Waterworks Company? A. That was about 1000*l.*, I think. — Q. Do you think it was that precise sum, or thereabouts? A. Thereabouts. — Q. It is not often so convenient as to be a round sum, but was it 1000*l.* or 1000*l.* odd? A. The whole put together was about 6000*l.*—not for that one estate. — Q. Not for the Waterworks Company? A. No, there was some property sold down near Bassaleg. — Q. We have heard to Sir Charles Morgan and Bailey. A. That is the railroad—that is some time ago. — Q. I understood you to say you had sold some property to the Water Company? A. I must recollect. — Q. And you also sold some property to the Railway Company? A. Yes, I was obliged to do so. — Q. We will confine ourselves at this moment to those two sales. Do you know what you got from the Waterworks Company? I got about 1000*l.*, I think, or thereabouts. — Q. Now, then, from the Railway Company—you tell us there were two sales there. Do you know what each of them were? A. Two or three, I think. — Q. Do you remember what the figure was that you were to have from the Railway Company? A. 2000*l.*, I think. — Q. Was it 2000*l.* for the whole or 2000*l.* for each? A. Not 2000*l.* for each, but 9000*l.* I think it was altogether. — Q. 9000*l.* altogether? A. Yes, for the three or four properties. — Q. We are told that you sold some of it to Sir Charles Morgan and Mr. Bailey? A. Yes, that was the Railway Company. — Q. I think not; I may be wrong. We are told you sold a bit, which is about a mile and a half from Newport or two miles, to Sir Charles Morgan. Do you remember anything about that? A. Yes, I

remember it perfectly well. — Q. Do you remember selling any either to Sir Charles Morgan or Mr. Bailey? A. Through the medium of my attorney. — Q. Mr. Haynes? A. Yes. — Q. Can you tell the sum at all that you were to get for it—you do not remember, do you? A. I do remember perfectly well, but I do not know that any one has a right to ask me these questions, because I am mistress of my own property, and it is not a common-place thing in the world to have your children to call you to account. — Q. I will not press you to answer, but these gentlemen are on their oaths, and I am bound to ask you the questions which they suggest.

A Juryman.—Do you think she understands the position we are in.

The Commissioner.—These gentlemen are under the order of the Lord Chancellor: they are summoned here. — A. To see my competency to answer their questions. — Q. Your competency to take care of your property, or whether some person should not look after you and your property; take care of you, and see that you are not imposed upon as to your health or property; that really is the object of those gentlemen. A. That I am not? — Q. They have not formed an opinion yet. I can do no more than suggest to you the propriety of answering the questions which are put to you. Your counsel, who are here, will check me if I do anything improper.

A Juryman.—That was the reason why I asked the ladies to leave the room, that you might speak more freely.

Mrs. Cumming.—I am under no intimidation at all from them, because they are intimate friends.

Foreman.—Your courtesy is very proper.

The Commissioner.—Have you executed any conveyances of these properties? have you signed any deeds? A. Of course, they could not have the property if I had not. — Q. Who brought in those deeds? who was your solicitor? A. Mr. Robert Haynes. — Q. Has Mr. Robert Haynes given you an account of all his receipts and payments to a recent time? A. Yes, he has. — Q. You would not like to show it to me? A. No. I would not mind showing it to you, or these gentlemen, but there is another party that I would not wish it to be known to. — Q. You say that a considerable part of the sums produced by these sales has been expended in law expenses? A. Exactly. — Q. Have they sent you a bill of those law expenses? A. About four or five thousand pounds or thereabouts. I do not tell you what I have had to live upon, because I had no other income but from my property. — Q. I want to see what has become of this which you told me just now was 9000*l.*, but which you and I, adding them up together, made only 7000*l.* You told me that the greater part has gone in law expenses? A. And so it has. — Q. Has Mr. Haynes ever sent you an account, because they are bound to give you a written account? A. Yes, he has; at least his brother did. — Q. Who is his brother? I did not know that he had a brother? A. Oh, yes, he has. There is Carlon and Haynes, besides. — Q. Carlon and Haynes have been your solicitors also? A. No, they were all at the same time. — Q. Do you have two sets of solicitors at the same time? A. My daughters, as I am forced to call them.—I was forced to raise money to go on. — Q. Why did your daughters force you to raise money to go on? A. Because I was forced to pay my lawyer's expenses. — Q. And you think these amounted to three or four thousand pounds? A. Yes; that is speaking within bounds. — Q. And have you had an account of all that? A. Yes. — Q. I do not know what it ought to be. I have no means of knowing. — Q. Who has been in the habit of hiring your servants from time to time? A. Myself. — Q. Do you get characters with them, for you seem to have been rather unlucky with your servants? A. I was very unlucky when Mr. John Ince used to send me servants—very unlucky. — Q. When did he send you servants? A. A good many times. — Q. When? A. Some years ago. — Q. Has he sent you any since I had the pleasure of seeing you at the Horns Tavern? A. Not to my knowledge. — Q. Has he sent any that you took in consequence of his sending them here? A. No, not exactly; he has sent them here to me without my knowing they were sent by him. — Q. You heard so afterwards? A. I did. — Q. Were they sent by Mr. Ince or Mrs. Ince? A. Mr. Ince. — Q. Not Mrs. Ince? A. Not that I know of. — Q. What makes you think that Mr. Ince sent you the servants? A. To suit his own convenience. — Q. If he sent them, he sent them for some purpose. But what makes you think the same from him more than from me? A. Because I do not think you would have done such a dirty trick. — Q. Why do you think he would do a dirty trick? A. Because he is accustomed to do dirty tricks. — Q. I do not like to condemn a man without cause? A. I do not like to ask you to condemn him. — Q. Could you tell me one or two dirty tricks he has done? A. In taking Captain Cumming away from his own house; he got him there, and took his writing desk, and overhauled all his papers, when the man was not fit to be removed. — Q. He took his writing desk with him? A. Yes, in a hackney coach. — Q. There were hackney coaches in those days; they are abolished now. Is there anything else you have to say against Mr. Ince? A. No, nothing worth mentioning. — Q. But you have had other things against him? A. Yes, a good many other things. — Q. Will you allow me to judge of them as well as yourself? Could you tell me one or two more? (No answer.) — Q. You could not show me any of Mr. Haynes' previous accounts before this one, which I hold in my hand? A. I should not like to do so unless Mr. Haynes were here. — Q. I will not press you. A. Because I think it would be very unladylike in me to do so. — Q. They are your own; you are quite at liberty to act without reference to Mr. Haynes. Mr. Haynes has no business to stop you from showing them if you like? A. He would show them himself if you wished. — Q. He formerly lived here, I understand? A. Yes. — Q. Now he is living at a short distance from you? A. Yes. — Q. He formerly lived here? A. Yes. — Q. Is his a better house than this? A. Yes, it is a larger house; but this is large enough for me. — Q. Have you ever said that Mr. Haynes is living in his present house on your money? A. No, but I have been told that others have said so; and amongst others, Catherine Ince has told every one about the neighbourhood that I am kept a prisoner, and that Mr. Robert Haynes is living upon my property. — Q. All the people here have that impression? A. I do not say they all have that impression. — Q. But Catherine Ince has told the people so? A. Yes. — Q. When I last had the pleasure of seeing you I was obliged to be very impertinent, and talked to you a good deal, but there was an arrangement come to. I don't ask you whether you were satisfied with the arrangement, but I speak to you about your solicitor and counsel at that time. Do you remember the purport of an arrangement? A. I remember that the property was my own, wholly and solely, to do what I liked with it. I believe that is correct, Sir. — Q. I must not give an opinion about that, but there was an arrangement made. Do you know why it was not carried out? A. Because I have no right to do it. I have the property in my own hands, and of course I would not, as I said at the office, consent to give up a whole loaf and take half. — Q. But were you not a free agent when you entered into that arrangement? A. I was brought there for the purpose of inquiring into my capacity, and whether I knew how to manage my affairs. — Q. And your solicitor and counsel entered into the same arrangement. I will not tell you whether in my opinion it was right or wrong, unless you ask me. If you did, I do not know that I should hesitate to give an opinion. But the arrangement was entered into? A. Yes. — Q. You were a free agent to enter into it? A. I was taken back to the madhouse, and so I could not be a free agent. — Q. Were you taken back to the madhouse? A. Yes, I was taken back again. — Q. The last day? A. It was the last day; but I believe you were the gentleman. — Q. I was. What was done with you afterwards, because you ought not to have been taken back, and my impression was, that you were not taken back. Do you remember where you went to on that day when we separated? A. I went to Mrs. Hutchinson's. — Q. Somewhere near Vauxhall Bridge? A. Yes. — Q. Do you know where you went to after that? A. I believe I went to Camberwell. — Q. And then you came here? A. And then I came here. — Q. That is what you think is the case? A. I am certain it was the case. — Q. To Mrs. Hutchinson's, near Vauxhall Bridge? A. Yes. — Q. And then you came here? A. No; I went to Camberwell. I had a small house at Camberwell, and there I remained till I was persecuted there again by my daughters. — Q. In what way did your daughters persecute you at Camberwell? A. By coming and intruding themselves always upon me. — Q. Can you give me an instance of what you mean by intruding upon you more than coming to your house? A. Yes, going about the neighbourhood and trying to injure my character, when I was at Camberwell. At the time I first came I was treated with every respect by the tradespeople, but after they had been there I found very different conduct, and I call that very injurious to any one. — Q. When you came here did you remain here some time? A. Yes, I am living here now. — Q. But you have been away in the interval? A. Yes, but I was not tied up to one place. I suppose many gentlemen and ladies do the same as me. — Q. Where did you go to next? A. I went to Worthing. — Q. No, I think you went to an intermediate place. Did you not go to Maida Vale West, Howley Villa? A. I went there; I did not stay long. — Q. What made you go away from here? A. I do not know; nothing particular. I tell you my daughters were eternally persecuting me. — Q. Was that your reason for going away from here? A. Yes. — Q. That your daughters bothered you here? A. Yes, and everywhere I went to. — Q. Did they come to you at Maida Vale West? A. Yes. — Q. Did they interfere with your neighbours here and there too? A. Yes, they did, here and there too. — Q. In Camberwell they got you in bad repute? A. Yes, they gave me the character that I did not pay any one; and that in London you know is very injurious. — Q. That was the reason why, you think, you went to Maida Vale? A. Yes. — Q. Now you have been back here some time, have they been doing the same thing lately here? A. They have called here. — Q. Since you have been here this time? A. I do not think they have. To tell you the truth, I do not think they have. — Q. When you were here, you went away a short time. Before you went away, they tell us some policemen came into the house one night? A. Yes, the house was full of policemen. — Q. I am in error, it was the other house. I am afraid I have misled you. I am given to error sometimes? A. Yes, but you are not called to account for it as I am. — Q. Yes, I am; and rather roughly sometimes. Now, at Maida Vale West do you remember the policemen coming in? A. Yes, I have reason to remember it. — Q. They say you were at the window? A. I could not get up to the window, it was too high. I could not lift the hasp of the window to open it. — Q. What made the policemen come in then? A. They were called in. — Q. Called in by the servants? A. Yes, they were sleeping in the house, and two navigators there. — Q. Do you mean the policemen were sleeping in the house? A. Yes, they were; the servants had them in every night. — Q. I do not want to doubt your word, but what makes you think these policemen were sleeping in the house? A. I am certain of it. — Q. Every night? A. I cannot exactly say every night; they were there frequently. — Q. Had you not respectable servants at that time? A. I thought they were. — Q. The coachman, was not he a respectable man? A. He did not sleep in the house then. — Q. Was he in the house that night? A. No, he was not in the house that night; he slept over the stables where the horses were. — Q. But he came in, and you saw him that night? A. But this was almost every night that they were there, and two navigators as well. — Q. And the policemen? A. Yes. — Q. Do you mean the same policemen that came in that night, or merely policemen generally? A. I cannot tell you that, for I was up in my room, and very ill. — Q. Did you not cry out at the window at all before the policemen came into your bed-room? A. I called out when the woman, Mary Hickey, I think her name was; no, Mary Rainy.

The Commissioner.—You cried out when she did what? A. When she was going to confine me with a strait waistcoat. — Q. Had she a strait waistcoat then? A. She made my shawl up as a strait waistcoat. — Q. What kind of shawl was it? A. It was a shawl. Mr. Haynes had the shawl to show to any one. — Q. Was it a green one? A. No, a white one. — Q. Was not there any colour in it at all? A. No, only dirt, if there was any colour at all.

A Juryman.—Was it a mottled worsted one? A. Yes. — Q. Was it one of your own making? A. No, it was not—it was a bought one.

The Commissioner.—This is your own house? A. Yes. — Q. But you do not wish to tell us exactly what you gave for it? A. You will excuse me that. — Q. Is the furniture your own? A. All my own. — Q. You bought it, everything as it is? A. No, not exactly so. I bought it, furniture and all; but there are a good many things I have put in myself. — Q. You tell me the cats were not in your bed-room always? A. No, they have come up and down. — Q. Are they in your house now? A. No, not all of them. — Q. Had you any at Brighton? A. No. — Q. Did you take the cats to Brighton at all? A. Yes, I did. — Q. We are told there were four or five? A. Ah, yes, seven, or eight, or ten; I dare say you were told. — Q. Now, the navigators: do you know who they were who were in the house? A. No, I do not. — Q. Who told you they were there; for I should like to inquire a little into that? A. It is necessary to inquire into it. — Q. It is not long ago, you know—it was last winter. A. Yes, it was. — Q. You took the house, I think, of Sir Matthew Wyatt? A. Yes. — Q. And there was a paper signed in his presence? A. Yes, Thorne, he went up to the office, and got the paper signed for and against, for one side and for the other. He is an old friend of Sir Matthew Wyatt's. — Q. But you were employing him at that time? A. Yes, but I did not know what the intimacy was between them at that time. — Q. Did you ever see Sir Matthew before? A. Before when? — Q. Before you went to take the house? A. No, I did not. — Q. You went to his house, I think, once? A. Once I went, but he was very poorly; at least, he had company there; there was a frivolous excuse made. — Q. And then he came to your own house, and signed the agreement? A. I think he signed the agreement up at the office. — Q. At whose office, Mr. Thorne's office? A. Mr. Thorne's office. — Q. You think you signed it there? A. No, I did not — Q. You think he signed it there? A. I think so; he was on very intimate terms with Sir Matthew. — Q. Were not Mr. Thorne, and you, and Sir Matthew, at the Villa when you signed it? A. He was at my villa then, but he was in the habit of going frequently to Sir Matthew's. — Q. You do not read the newspaper, do you? You did not see a letter that Sir Matthew put into the newspaper yesterday, did you? A. I have seen Sir Matthew's name in the newspaper. — Q. Did he pay you any particular attention? A. Me? — Q. Yes? A. What should he for? — Q. Because I was told that you thought him a very courteous man. Do you know that he is a married man? A. I do not know what he is, and do not care. — Q. Did he ever send you any game? A. He never sent me any, unless he has sent it to my servants, and that I cannot tell. — Q. You mentioned a little while ago, something about a will, and that Mr. Thorne had it? A. Yes, so he had. — Q. I am going to ask a still more impertinent question than I did before. Have you ever made a will? A. I did. — Q. When, do you know? A. The time I thought I was dying. — Q. When was that? A. After I had been at the madhouse. — Q. Since 1846—since you saw me? A. Yes. — Q. Can you tell me when about it was, when you made it? A. These things are so long ago, and never feeling in my own breast that any one would have a right to call me to account. — Q. You are of a certain age; you cannot tell about when it was—who made it for you? A. Mr. Robert Haynes. — Q. He made it? A. He made it, by my dictating it to him. — Q. You executed it—did you ever sign that will? A. No, it was not signed. — Q. You do not think it was ever signed? A. I do not think it, but I know it. — Q. You dictated it to Mr. Haynes what you wished it to be? This paper was handed to us as a paper which was handed over by you to Mr. Thorne. Do you remember whether this was the paper? A. I never gave it to Mr. Thorne. — Q. I thought you gave it to Mr. Thorne? A. No; I never had such an opinion of him, after seeing him once or twice. — Q. Very likely I am wrong. I believe I am. But in that paper, in the will you so directed Mr. Haynes to draw up, do you know to whom? You had not signed it, and therefore there is not the same delicacy in asking the question—do you know to whom you gave your money principally? A. Yes. — Q. To whom—was Mr. Haynes to have any? A. He was to have some of it. — Q. Do you remember how much? A. There was some to him, and some to his wife. — Q. And to anybody else connected with him? A. At the time I was extremely ill, when it was done—very, sir. — Q. And you cannot tell me when it was? A. I think it was when I was down at Vauxhall. — Q. Do you mean while you were living with Mr. Hutchinson? A. Yes. — Q. Directly after I had the pleasure of seeing you? A. Yes. — Q. And you told him to make it? A. I did. — Q. Was anybody present? A. No, there was not; there was a witness to it, you know. — Q. But you never signed it? A. I never signed it. — Q. Was anybody present when you told him what your wishes were? A. No, only him and me. — Q. Now, I am going to ask you another impertinent question, behind his back, but I must ask it. Did he make any objection to it? A. No, he did not; not to my knowledge. I do not know what he did behind my back. — Q. You told him to make the will; and when you gave him instructions about it, there was nobody present but you and him. Then I ask, in fairness to you, whether he made any remonstrance about the way in which you were disposing of your property? A. No; he put as little questions to me as he possibly could; for he saw I was such an invalid, that I was not able to answer him. — Q. Did he ask you whether you wished to leave anything to your own family? A. No, he said nothing. — Q. Did he express any wish that you should not insert his name in the will? A. No. — Q. You do not remember that? A. No. — Q. I will not trouble you to read this, because it is a long averment; but the result of this is to give a considerable sum.

A Juryman.—How much?

The Commissioner.—Do you remember how much you were to give to Mrs. Haynes? Some, you say, to Mr. Haynes, and some to Mrs. Haynes? A. But that is gone now, you know; that is burnt, that will is. — Q. But when you gave Mr. Haynes, at the Gas Works, at Vauxhall, directions to make the will, do you remember how much you had given him? A. That I cannot tell exactly, without referring to it. — Q. You say that you burnt the will itself? A. I burnt it. — Q. Did you ever sign it? A. No. — Q. Have you ever made any other will since 1846? A. No, I have not. — Q. Then you cannot tell us at all what figure you ascertained for Mr. Haynes or Mrs. Haynes? A. No. — Q. Or the children? There was some for Mrs. Haynes' children, was there not? A. I believe so, but it is totally out of my memory.

A JURYMAN.—Was there anything to take concerning Mrs. Hutchinson? A. No, I don't think. — Q. Do you remember whether you told him to give anything to Mr. or Mrs. Hutchinson? No, I do not recollect it.— Q. Or to Miss Hunt, who, we were told, was kind and attentive to you? A. Yes, there was something to her, but that is all obliterated—it is burnt. — Q. Did you ever sign it before it was burnt? A. No, I never did.

A JURYMAN.—Is there any will in existence now? A. No, none.

The COMMISSIONER.—No written document at all? A. No.

A JURYMAN.—Did she know anything of her father's will?

The COMMISSIONER.—Have you any recollection of your father's disposition of his property? I may ask you that, because that is a public document. Do you recollect? A. I could not take the liberty of asking about it. — Q. But your father is dead, and he left a will, which I may read in Doctors Commons, upon the payment of a shilling, I can go and see it? A. I know you can. — Q. Do you know the contents of his will at all, because you were interested in it? A. Yes; but I do not know that any one else is interested in it. — Q. If I am told rightly, your daughters were interested in it? A. No, sir. — Q. I tell you fairly that I have not read it, but I am told that your daughters are interested in it? A. No. — Q. I am told that it was not a great deal that was left under that will? A. Not a great deal. — Q. We may differ about what is a great deal. From this, I should say it was between two and three thousand pounds? A. Do they say that that was all my father's property? — Q. I do not know that it is; but it appears that there is a sum of 2,800*l.* and odd, which is directed to be paid, the income of which is directed to be paid in moieties to Mrs. Hooper and the Inces. Do you remember the contents of your father's will? A. No, I do not; but I know that is a falsity, whoever put it in, because that was not my father's will. — Q. Did not your father leave some of the property to you for life, and then to your two children? A. He does not mention the children in the will. — Q. He does not? A. No, he does not. He leaves it to me wholly and solely, and that it should not be liable to my husband's debts.

*Dr. Caldwell* (To the Commissioner). I think, sir, she has been long enough under examination. I think she is somewhat confused.

Mr. PETERSDORFF.—I do not think she is too fatigued.

The COMMISSIONER.—In 1848, you must have had your attention drawn to your father's will. Do you remember a chancery suit in 1848? A. What chancery suit? — Q. A suit about your father's property? A. Yes, but I did not know anything about it. I did not take the liberty of asking my father.

The COMMISSIONER.—He was dead in 1848—was he not? A. He died after he had made a will. — Q. He was not alive when I had the pleasure of seeing you at the Horns Tavern? A. No; if he had been alive, I never should have been taken there. He never would have suffered his grandchildren to treat their mother as they have treated me. — Q. You went from Maida Vale West to the Edgeware Road—did you not? A. Yes; to Sir Matthew Wyatt's house. — Q. That was Howley Villa—Sir Matthew Wyatt's? A. Yes. — Q. Where did you go when you went from Sir Matthew Wyatt's house? A. I went down to Worthing. — Q. I think there was an intermediate place? A. On the road side there was a little place. — Q. Was it the Edgeware Road —do you remember being in the Edgeware Road? A. That place you allude to was not in the Edgeware Road, it was many miles from London—a good many miles from London. — Q. I think you went from Maida Vale West, from Howley Villa to Stamford Street? A. Yes. — Q. Who lived there? A. Mr. and Mrs. Hutchinson. — Q. From there, did you not go to the Edgeware Road? A. Yes, I did; but I was hunted from there by my daughters. — Q. From Stamford Street, you were again hunted away? A. Yes, I was. — Q. Could not Mr. and Mrs. Hutchinson prevent your being hunted? A. Yes; and so they did until their house was broken into, and that fellow, Ebenezer Jones ——. — Q. I was going to ask you a question about him presently—have you seen him? A. I saw him in 1846.—Q. You know he was one of the witnesses there? A. Exactly—FOR ME. — Q. You and I cross-examine one another: I wanted to know about a little transaction that took place in the Edgeware Road, about a year ago, was it not—whose house were you in there—do you remember the name of the person?

A JURYMAN.—Oldfield.

The Commissioner.—Mrs. Oldfield? A. Mr. Oldfield and Mrs. Oldfield.— Q. Who took that house for you? A. I went there, and took it myself. — Q. Were you mistress of the house? A. No. — Q. They were lodgings—do you remember what rooms you had there? A. Yes, perfectly well. — Q. What were they? A. A drawing-room and bed-room. — Q. Did not Mrs. Ince come and see you there? A. Yes, she did come and see me there—she pushed the servant almost down stairs to pounce upon me. — Q. Do you remember how often she came? A. Yes, three or four times. — Q. Do you remember what time of the day it was? A. It was when I was taking my lunch. — Q. Did you say the lunch was very cold? A. Had I got a cold? — Q. Did you say the lunch that was on the table had got cold, and that you could not help yourself to it? A. No, never: if anybody says I said so, they have told a falsity, and I would tell them so to their face. — Q. If Mrs. Ince said it? A. Mrs. Ince is very capable, I am sorry to say, of saying anything but the truth. — Q. I want to speak to you a little about Mrs. Ince presently? A. The less the better, if you please. — Q. I do not like to touch upon that, because I was told it was a delicate subject, but you will excuse me, presently, if I ask you one or two questions—she saw you one day, and then came the following day—did she not? A. She was continually there. — Q. Did you see her, do you think, more than twice there? A. No, I did not. — Q. Do you remember how long she stayed the first time she came? A. I could see her in the street, through the window of my drawing-room, surrounded by policemen, and a set of vagabonds round her pointing to the house—that is lady-like conduct. — Q. Are you quite sure you saw policemen about her? A. Yes; I saw her speaking to the policemen. — Q. Who do you mean by vagabonds? A. Not very respectable looking people. — Q. Are you quite sure it was her? A. Yes, I am sure it was her. — Q. Was it once, or more than once? A. More than once—several times. — Q. The first day she was two or three hours, was she not? A. I had no watch to look at. — Q. Did she dine with you? A. No; she never dined with me. — Q. Nor drank tea? A. No; I did not ask her to tea—she came when I was taking my lunch. — Q. And she stayed there? A. Yes. — Q. Some hours? A. Yes. — Q. Was Mr. Haynes there too? A. No, he was not first of all, but he was called afterwards. — Q. Did you send for him? A. I sent for him, and I sent for Mrs. Oldfield, and Mr. Oldfield came. — Q. Then Mrs. Ince called on you a third day, she tells us, whether truly or not, I cannot pretend to say? A. I cannot tell. — Q. Did you forbid her coming in again? A. I was displeased at her intruding herself upon me on those two occasions. I never saw her afterwards. — Q. Do you remember forbidding her to come in? A. She did not say she was coming in; she bolted in upon me, and put her arms round my neck. — Q. It was upon that occasion? A. That was the occasion when the proceedings were going on against me in court; issued out by Mr. John Ince, her husband. — Q. Did you expect her to come the following day? A. No, she was quite unexpected; she brushed up, and nearly pushed the servant down stairs. — Q. Did you give any notice that if she did come, she was not to be admitted? A. After obtruding herself upon me as she did. — Q. Do you remember signing a memorandum, forbidding her to come in, and that there was a chain to the door? A. Yes, because she obtruded herself upon me, and kicked up such a row that it made the house quite scandalous. — Q. In the house? A. In the house, and out of the house, and Mrs. Hooper was along with her. — Q. Was Mrs. Hooper in the house? A. I do not know. — Q. Was Mrs. Hooper in the house in your presence? A. Never. — Q. Now, as to Mrs. Ince herself, I think, from what you tell me, that you do not seem to have a very good opinion of her? A. I have not a good opinion of her. I never said anything about her. — Q. You say she was with these people outside the house? A. I say it was not a proper place for a person calling herself a gentlewoman to be surrounded by a parcel of policemen. — Q. You are quite satisfied you saw her about the house? A. Quite. — Q. You say you had an impression that she had once attempted to strangle you? A. That is the one I allude to—that is the time when I tell you she came and put her arms round my neck. — Q. When was that? A. When I was at Oldfield's. Of course, any one who came to see me would knock at the door first. — Q. She came in there without knocking at the door? A. Oh, yes. — Q. Are you sure that took place at Oldfield's? A. Yes. — Q. Did she at any time make any attempt to strangle you? A. I do not say she attempted to strangle me; though that is what it is said I did say.— Q. What did you say—we will hear it from yourself—what did you say about it—we may be misled? A. I was very much frightened when she came into the room, throwing the door open, running up to me, putting her arms round my neck, after the statement received that Ince and her were taking proceedings against me in court. Now that is a very strange thing if you are taking proceedings against a person to come in a very cordial way. — Q. Why should you not put the best interpretation upon it, and suppose that it was an act of affection, instead of anything else ? A. Affection, sir ! — Q. It is my duty not to set you against her, or her against you: what makes you think it was not an act of affection ? A. Of course, you would not take proceedings against a person if you were partial to them. — Q. That depends on whether it is necessary or not. What proceedings had she taken, then, besides the original commission ? A. Her husband, I suppose it was, or both of them, instituted proceedings against me. — Q. For what ? A. To get me into a madhouse, or to get my property. — Q. Was there any other occasion on which you think she did anything of that kind which you consider something like strangling, or that unkind act ? A. I never mentioned that she strangled me, or wanted to strangle me; but I said, and say again, it is a very strange way of behaviour, while taking proceedings in the court against me. — Q. I do not understand why there was not an end of all the proceedings after 1846 ? A. I understood so : I was proclaimed in court a free agent, to do as I liked. — Q. Do you consider that you had been a free agent from that time to this ? A. I considered it when I left the court. — Q. Have you been a free agent from that time to this. You have been changing your house very often ? (No answer.) — Q. Mrs. Hooper was your eldest daughter, I think ? A. Yes. — Q. She married a person ? A. In the band. — Q. A person who was once in the band, and you thought, perhaps naturally, that it was not so good a match as she ought to have made ? A. No : perhaps some people would be satisfied with it. — Q. She seems to have had some children; was there not a reconciliation after that: she married in 1836 ? A. I paid a good many of their debts after that. — Q. In 1839, or two or three years after she was married, was there not a reconciliation between you and her ? A. We were never on the terms that we were on before. — Q. Did she not come and stay at your house ? A. Her child was ill, and her medical man attended the child. — Q. After the birth of the second child, in 1839, Mr. and Mrs. Hooper came and stayed with you for different periods—did they not ? — A. She did with the child. — Q. Did not Mr. Hooper ? A. No, he did not. — Q. He never was in the house ? A. Yes, he was in the house, and had dinner. — Q. Was it not a perfect reconciliation at that time ? A. It was on her side a necessary one. — Q. But not on yours. Has she behaved ill to you in any other way besides this marriage, which you think unfortunate ? A. Perhaps these gentlemen might not think it so at all. — Q. Has she behaved in a ny way to give you offence independent of that? A. I have never been reconciled since that, and what is more, never shall. — Q. Let us hope you will some day or other? A. Never—never. — Q. Never too late, you know ? A. To mend. — Q. Were you ever under an impression that either of your daughters had made an attempt to murder you ? A. No ; I never stated it. — Q. Had you the impression on your mind at any time ? A. No. — Q. Or that they had attempted to poison you ? A. No; but, as I said before, there was stuff put into the food for the fowls—at least, not put for the fowls, but put into some food, and was given to the fowls ; the next morning, one of them was found dead. — Q. What was it put into? A. Into the food. — Q. What food was it ? A. Oatmeal and barley. — Q. Who did it; do you know ? A. That I cannot tell you. — Q. Do you know who did it ? A. No, I do not. — Q. You have never accused anybody of it ? (No answer.) — Q. You say it was put in the oatmeal ? A. Put into the fowls' victuals. — Q. But that could not be with the intention of poisoning you ? A. No, it was not intended to give it to the fowls ; but you know that the cats would not touch it—that it was thrown out to the hen roost—it was not thrown about the place, because fowls will pick up anything—then, of course, you could not. — Q. Then it was given to the cats, and became the food of the fowls ? A. Exactly. — Q. What makes you think it was done to poison either you or your cats ? A. I don't say so ; but it was not a delusion of mine, because there was Dr. Barnes analyzed it. — Q. There was the stuff at all events ? A. There was the stuff found. — Q. And what was found? A. It was found in a jug where the milk was—there was sugar of lead in the stuff, but Dr. Barnes can explain that to you. — Q. Do you remember what it was? A. I do not remember what it was—oxalic acid—Epsom salts was put into the milk. — Q. That was a different occasion? A. No, it was that same time—the milk was thrown out—the servants had put some salts into a jug, and never washed it out, and the milk was put in—now understand me. — Q. Was it milk or cream ? A. It was milk. — Q. Not cream ? A. No, it was not cream ; it was milk. I had sometimes cream, and sometimes milk. — Q. Your daughters would have nothing to do with that? A. I did not say my daughters had. — Q. They would have nothing to do with that? A. How it came there I do not know; but there it was. I do not wish any one to take my word for it. — Q. But your daughters would have nothing to do with that? A. I do not say they had. — Q. I want to exculpate them.

A Juryman (To the Commissioner).—I do not yet understand about the milk that was thrown away. She speaks of milk and oatmeal; was that milk and oatmeal too?

The Commissioner.—Did this happen more than once? A. No, only once; and I sent it to the chymist to be analyzed, and he said it was oxalic acid and Epsom salts. The jug had Epsom salts in it, and the milk was put into that very jug. — Q. And then was that given to the cats? A. It was offered to them. — Q. But they would not drink it? A. No, they would not. — Q. Then it was thrown away? A. It was thrown away. — Q. And the fowls eat it? A. And the fowls eat it. — Q. The stuff that was in the jug would have melted in the milk, and would have got thrown away in that way, would it not? A. Yes. — Q. There were two kinds of things? A. Yes; there was one in the milk, and the other was in the food for the fowls.

A Juryman (to Mrs. Cumming).—Are you fatigued? A. I am very much fatigued.

The Commissioner.—We will go away now, and come back again in half-an-hour, if you like? A. You are very good. — Q. These gentlemen would like a few more questions to be asked. Could you tell me the annual amount of your property in Wales? A. Yes. — Q. Do you know what it is now? A. Yes. — Q. What is it now? A. The same as it was then. Do not think this an impertinent or short answer. — Q. Certainly not. You say it is now what it was? A. I do not say what it is now. — Q. You say it is now what it was then? A. It is the same. They insinuate that I have been squandering the money and property. — Q. I do not know? A. But I know. — Q. I think you put it rather strangely? A. Stronger language. — Q. I do not mean anything offensive. What do you think is your income from the property at Newport? A. The property altogether, do you mean? — Q. Yes. A. About 400*l.* or 500*l.* a year. — Q. Is it that now? A. It is that now, and it was that. — Q. It was that in 1846? A. Yes. If I had been squandering my property, how could I have the same income? — Q. But you have sold some of your property? A. Yes, but I have improved it. — Q. Have you laid out any money in repairs? A. I have put the farms all in repair. — Q. There is one celebrated name, the Bird's Nest, are the others in good repair? A. Yes, exactly, and so are all the others. — Q. Do you know how much was laid out on the Blackbird's Nest—that is a prettier name than the other—within the last year or two: your accounts will show, I suppose? A. Yes. — Q. Do you remember how much? A. I have got an account of it, because he knew we paid all the bills as they were furnished. — Q. You have not been down there yourself? A. Yes, I have. — Q. Since 1846? A. I went to the Blackbird's Nest. — Q. From 1846? A. Yes, I have. — Q. I think I asked you just now, from place to place, where you had been, and I don't think you mentioned that?

Mr. Southgate.—She was there all the summer.

A Juryman.—Has not your property increased since you sold to the Railway Company and the Water Works Company? A. The value of it has increased. — Q. Then that makes it more than five or six hundred a year? A. No, I beg your pardon.

The Commissioner.—You have more than one tenant, of course? A. I have a good many tenants. — Q. Who is the man who pays you the greatest rent? A. Blackbird's Nest, I think. — Q. That is the highest figure, is it? A. I think it is. — Q. Do you know what it is? A. I think I do. — Q. Do you remember what it is, the Blackbird's Nest? I suppose you have an account rendered from time to time? A. Yes; I have been down twice since that time, and received the rents myself. — Q. Do you know what the Blackbird's Nest was? A. Seventy pounds a year, or thereabouts. If I had my books, I could have told you immediately.

The Commissioner.—The autumn of 1846. Do you remember seeing Mrs. Ince at the Horns Tavern on that occasion? A. I do, and Mrs. Hooper too. — Q. Are you quite sure Mrs. Hooper was there? A. Yes, she was in the room. — Q. In the jury-room, where I was? A. Yes. — Q. Are you quite sure that you saw Mrs. Hooper? Did you see Mrs. Ince there? A. Mrs. Ince was always there. — Q. And Mrs. Hooper? A. And Mrs. Hooper. — Q. I thought Mrs. Hooper was ill at the time? A. So it was reported. — Q. Do you think she was in the room? I understand ill? A. I was ill, but I was brought there. — Q. Did you see Mrs. Hooper in the room? A. I think she was there, but she never came up against me. — Q. You saw her sitting down? A. I think so. I could not say that it was Mrs. Hooper, but it was very like her at the distance she was from me. — Q. Did you see either of them at the bar of the Horns Tavern? A. No, I did not; but I was told they were both at the bar. — Q. But you did not see them? A. I did not, for I do not go to those kind of places. — Q. I doubt very much whether she was there; have you any reason to suppose that Mrs. Ince was drunk there? A. I never said she was. — Q. Were you told so? A. I was told she was at the bar. — Q. Were you told that she was drunk at the bar? A. No. — Q. But that she was at the bar? A. That she was at the bar. — Q. I do not pretend to say whether she was or was not, but from her appearance, I should not think it likely? A. You must not judge always from appearances. If a lady is seen in the street, with a parcel of policemen talking to her, and pointing up to the house, that is not very like a lady. — Q. But are you quite satisfied she did that? A. I saw it myself. — Q. That was in the Edgeware Road? A. Yes. — Q. Mr. Ince, we understand, lost two of his children? A. I do not know how many he has lost. — Q. Do you remember seeing one? A. I saw one of Mr. Ince's, and one of Mr. Hooper's children. — Q. Was there anything peculiar about one of Mr. Ince's children? A. I never made any remarks. — Q. What did you say? A. When I saw the child, I said it looked a very pretty corpse: that was the expression I made use of; and as to Mr. Ince's child, I said it was very much emaciated; and so it was—it had suffered a great deal before his death. — Q. Was there not one that you said was glazed? A. Oh, no! I cannot help smiling at that. — Q. Did you ever say that? A. No—so help me God! — Q. Have you ever said it was like a waxen doll in a tailor's shop, or any phrase of that kind? A. No, I have not, but it has been said so. I heard it myself—was told it. — Q. But you never said it? A. On my oath. — Q. But I do not put you on your oath, you know?

A Juryman (to the Commissioner).—She told you she had seen a book.

The Commissioner.—I suppose you have have got a rent-roll? A. Of course I have. — Q. Will you show it to me? A. You must excuse my doing that, as the commission is held upon my understanding, and upon the validity of what I did, and that it would be probably said I am imbecile to expose my private affairs. — Q. Now, as to Mr. Ince, in what way had he ever behaved ill to you? A. Oh, sir! — Q. Can you give me any instance of it? A. Yes; I could give you many instances, if I was well enough. — Q. Would you like us to go away, and come back again in half-an-hour? A. I am quite exhausted. — Q. You told me, a little while ago, you were at Worthing? A. Yes, that is true. — Q. Do you know what name you went by? Did you go by your own name? A. No, I did not. — Q. I am not finding fault with you, but did you go by any other name there? A. Yes, I did, that I might not be found out by my daughters; that is the truth. — Q. Was it Cunningham? A. No, Cleveland.

A Juryman.—We are here as kind friends to you; you may tell us any secrets you like, because we are friends of yours.

The Commissioner.—You never pay any interest upon the mortgage, do you? A No, I never was applied to. — Q. You do not know what it is? A. No. — Q. Who told you that Mrs. Ince was at the bar of the Horns Tavern? It was not me? A. No; and if it was, I would never say it was you; it would be unhandsome, if a friend tells you; it would be unladylike to mention it. — Q. Somebody told you so? A. Somebody told me so. — Q. Did you inquire whether it was founded on fact? A. No, I did not; I was so much hurt at it, that I did not. — Q. I wish you had made inquiries, to know whether she was or not? A. I did not, for I was very tired when I went away, and much excited. — Q. Doctor Caldwell says perhaps you would like a glass of wine, or a glass of brandy and water, is that so? A. No. — Q. Have you dined? A. No, I have not had anything to-day. — Q. I am afraid your appetite is not always very good? A. No. — Q. You will have nothing to drink? A. No, nothing to drink. — Q. You like something to drink better than something to eat, do you not? A. No. — Q. Your appetite is bad? A. Yes, it is. — Q. What time will you dine to-day? A. Oh, any time I can. I have two ladies here, you know.

The Commissioner.—Do they dine with you every day? A. Very often they do, one or the other.

A Juryman.—But you have no particular hour? A. Five o'clock.

The Commissioner (rising).—Good morning.

Mrs. Cumming.—Good morning.

The Commissioner.—I am afraid I have given you a great deal of trouble.

Mrs. Cumming.—I am afraid I have given you a great deal of trouble.

The Commissioner.—That does not signify. If we wish to see you again, we will let you know. You will certainly see me again; and if these gentlemen wish to see you again, probably you will have no objection?

Mrs. Cumming.—If I am able.

The Commissioner.—You will be able. Good morning.

Mrs. Cumming.—Good morning.

The Commissioner.—We should not have come to see you if we could have helped it.

Mrs. Cumming.—No, sir.

